# Two New Alginate Lyases of PL7 and PL6 Families from Polysaccharide-Degrading Bacterium *Formosa algae* KMM 3553^T^: Structure, Properties, and Products Analysis

**DOI:** 10.3390/md18020130

**Published:** 2020-02-24

**Authors:** Alexey Belik, Artem Silchenko, Olesya Malyarenko, Anton Rasin, Marina Kiseleva, Mikhail Kusaykin, Svetlana Ermakova

**Affiliations:** G.B. Elyakov Pacific Institute of Bioorganic Chemistry, Laboratory of Enzyme Chemistry, Far-Eastern Branch of the Russian Academy of Sciences, 690022, Vladivostok, 159, Prospect 100-let Vladivostoku, Russia; belik_a_a@mail.ru (A.B.); artem.silchencko@yandex.ru (A.S.); vishchuk@mail.ru (O.M.); abrus__54@mail.ru (A.R.); mikiseleva@mail.ru (M.K.);

**Keywords:** alginate lyase, enzyme specificity, polysaccharide, oligosaccharide, anticancer activity

## Abstract

A bifunctional alginate lyase (ALFA3) and mannuronate-specific alginate lyase (ALFA4) genes were found in the genome of polysaccharide-degrading marine bacterium *Formosa algae* KMM 3553^T^. They were classified to PL7 and PL6 polysaccharide lyases families and expressed in *E. coli*. The recombinant ALFA3 appeared to be active both on mannuronate- and guluronate-enriched alginates, as well as pure sodium mannuronate. For all substrates, optimum conditions were pH 6.0 and 35 °C; Km was 0.12 ± 0.01 mg/mL, and half-inactivation time was 30 min at 42 °C. Recombinant ALFA4 was active predominately on pure sodium mannuronate, with optimum pH 8.0 and temperature 30 °C, Km was 3.01 ± 0.05 mg/mL. It was stable up to 30 °C; half-inactivation time was 1 h 40 min at 37 °C. ^1^H NMR analysis showed that ALFA3 degraded mannuronate and mannuronate-guluronate blocks, while ALFA4 degraded only mannuronate blocks, producing mainly disaccharides. Products of digestion of pure sodium mannuronate by ALFA3 at 200 µg/mL inhibited anchorage-independent colony formation of human melanoma cells SK-MEL-5, SK-MEL-28, and RPMI-7951 up to 17% stronger compared to native polymannuronate. This fact supports previous data and suggests that mannuronate oligosaccharides may be useful for synergic tumor therapy.

## 1. Introduction

Alginate lyases are among the most studied carbohydrate-modifying enzymes. They are produced by organisms, which use alginic acids as a source of energy, and by alginate-producing bacteria to form exo-polysaccharide (EPS) as the main part of biofilms [1]. Their mechanism of action is based on β-elimination, i.e., cleavage of a bond between two β-d-mannuronic or α-L-guluronic links with the formation of 4-deoxy-L-erythro-hex-4-enopyranosyluronate (Δ) [1]. 

According to classical nomenclature, alginate lyases are divided into M-specific (EC 4.2.2.3) and G-specific (EC 4.2.2.11), however, there are also lyases with wide specificity that cleave bonds between М- and G- residues. They also have different types of action (exo- or endo-), and alginate oligosaccharide lyases (4.2.2.26) have the exo- type of action. Not all alginate lyases digest acetylated substrate [2], so the screening for this ability is an important step in the development of new antibiofilm tools. The optimum pH for these enzymes ranges from 5 to 10, and the optimum temperature ranges from 20 to 70 °С. Km value extends from 0.0026 mM on acetylated alginate *(Pseudomonas aeruginosa* strain PDO486 (DP 263) [3]) to 6.7 mM on polymannuronate from Qingdao BZ Oligo Biotech Co., Ltd. (China) [4].

By the amino acid sequence homology, alginate lyases can be divided into several structural families of polysaccharide lyases (PL) (http://www.cazy.com): PL5, PL6, PL7, PL14, PL15, PL17 and PL18 [5,6]. Most of the alginate lyases with different specificity belong to PL7, which consists of a β-jellyroll that forms catalytic cleft and three adjacent β-sheets with catalytic amino acid residues [7]. Catalytic domains of PL14 and PL18 also have β-jellyroll fold, while PL6 has a β-helix fold, and PL5, PL15, PL17 have (α/α)_n_ toroid folds [8].

Products of action of alginate lyases reveal multiple biological activities, presumably, due to their evenly distributed negative charge and ability to strongly bind with a protein molecule, modifying its conformation. For example, those obtained by *Pseudoalteromonas* sp. (DP 3–9) stimulate the immune system, increasing the production of cytokines (G-CSF, TNF-α, interleukins) in mural macrophages. The most active are unsaturated guluronate G8 and unsaturated mannuronate M7 oligomers [9,10]. Products of action of alginate lyase from *Agarivorans* sp. L11 (only DP 5) inhibit the growth of human bone cancer cells MG-63 by 60–70% [11]. Products of action of alginate lyase from *Sphingobacterium* sp. (DP 2–4) show antioxidative properties *in vitro* [12]. Alginate oligosaccharides inhibit growth and differentiation of adipocytes and absorption of saturated fatty acids [13,14] and exhibit anti-allergic properties by suppression of IgE [15]. 

Computer analysis of the distribution of alginate lyases and alginate lyase-encoding genes among different groups of living organisms reveals that there is still a great potential in experimental characterization of amino acid sequences with potential alginate degrading activity. There are more than 50 thousand known amino acid sequences, identified as alginate lyases, and 47 thousand of them belong to bacterial genomes. Just 260 enzymes have been functionally characterized: 181 from EC 4.2.2.3, 70 from EC 4.2.2.11 and 9 from EC 4.2.2.26. According to the CAZY database [6], amino acid sequences and functional characteristics are known for 27 alginate lyases.

Thus, the class of alginate lyases is still insufficiently studied, while its practical application is of high importance. Polysaccharide-degrading marine bacterium *Formosa algae* KMM 3553^T^ (*F. algae*) is one of the potential sources of genetic material for the expression of alginate lyases; these bacteria inhabit thalli of brown algae *Fucus evanescens* in the cold waters of Sea of Okhotsk [16]. 

The strain has been characterized previously as the producer of various carbohydrate-degrading enzymes: fucoidanases FFA1 [17], FFA2 [18], and endo-1,3-β-D-glucanase GFA [19]. Accordingly, *F. algae* can be described as a psychrotolerant organism, environmentally adapted and evolutionally specialized for inhabiting and utilizing brown algae, and containing enzymes, that are active at low temperatures. On the other hand, its optimum temperature of growth is 23 °C, so there can be other enzymes, stable at room temperature, that can be used in biotechnology.

In this study, we report on the progress of research on *F. algae*’s enzymatic machinery for biotechnology applications and the discovery of new alginate lyases ALFA3 and ALFA4.

## 2. Results

The yield of the target enzyme was 72 mg/L of cultural media for ALFA3 and 12 mg/L for ALFA4. Molecular weights, structural families of amino acid sequences, and substrate specificity are presented in Table 1.

**Figure 1:**
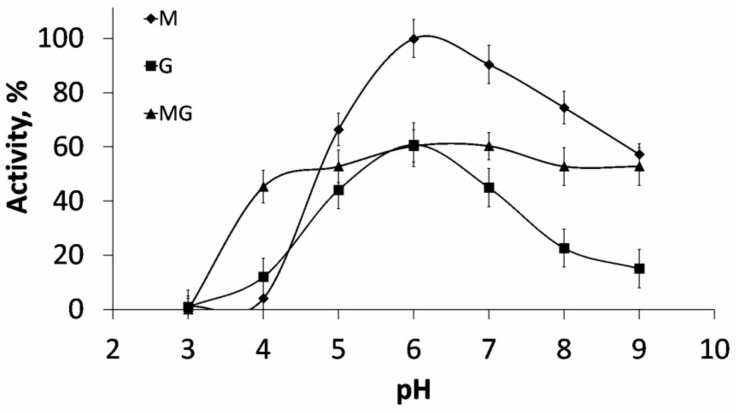
Optimum pH of ALFA3 when acting on different substrates. M – sodium mannuronate, MG – mannuronate-enriched sodium alginate, G – guluronate-enriched sodium alginate. Standard deviations are given.

ALFA3 digested three types of sodium alginates (M-enriched, G-enriched and MG-mixed) with equal effectiveness: 21.5 U/mg for M- and MG- and 18.7 U/mg for G-. The detected difference indicated that this enzyme belonged to EC 4.2.2.3 (mannuronate-specific alginate lyase). Optimum pH was 6.0 for all types of substrates (Figure 1).

The optimum temperature of ALFA3 was 35 °C (Supplementary Figure S1), half-inactivation time was 1 h 30 min at 42 °C. Ions of K^+^ and Ca^2+^ in concentrations up to 0.5 M did not influence the activity. Km was 0.12 ± 0.01 mg/mL (Figure S2). ALFA3 Vmax values were 0.128 × 10^−3^ M/min for G, 0.150 × 10^−3^ M/min for MG, and 0.211 × 10^−3^ M/min for M. Kcat values were 3.52 s^−1^ for G, 4.13 s^−1^ for MG and 5.80 s^−1^ for M.

Alginate lyase ALFA4 had the optimum temperature of 30 °C (Supplementary Figure S3), optimum pH was 8.0, the optimum concentration of NaCl was 0.6 M, half-inactivation time at 37 °C was 1 h 40 min. Km was 3.01 ± 0.05 mg/mL (Figure S4) of the mannuronate-enriched substrate. Vmax was 0.314 × 10^-3^ M/min for MG. Kcat value for MG was 2.88 s^−1^.

According to classification based on amino acid sequence, ALFA3 was determined to belong to PL7 polysaccharide lyase family and ALFA4 to PL6 family. As long as ALFA3 and ALFA4 digested mannuronate-enriched substrate more rapidly than guluronate-enriched, they were classified as mannuronate-specific (EC 4.2.2.3)

**Figure 2:**
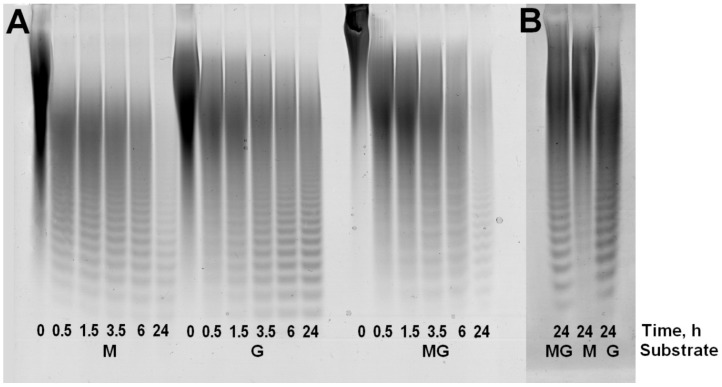
Kinetic analysis of digestion of different substrates by ALFA3 (**A**) and final products of digestion of different substrates by ALFA4 (**B**). M – sodium polymannuronate, MG – mannuronate-enriched sodium alginate, G – guluronate-enriched sodium alginate. Time of reaction is given in h. Each lane was loaded with 60 µg of sample.

Kinetic analysis of substrate digestion for ALFA3revealed that the depth of substrate cleavage strongly depended on M/G ratio: while sodium polymannuronate was completely digested to oligosaccharides in 24h (hydrolysis depth 88.3%), guluronate-enriched substrate contained a considerable high-molecular fraction (hydrolysis depth 67.9%), while this fraction was much smaller for the mannuronate-enriched substrate (hydrolysis depth 87.3%) (Figure 2). These fractions are not hydrolyzed even after addition of more enzyme. 

**Figure 3:**
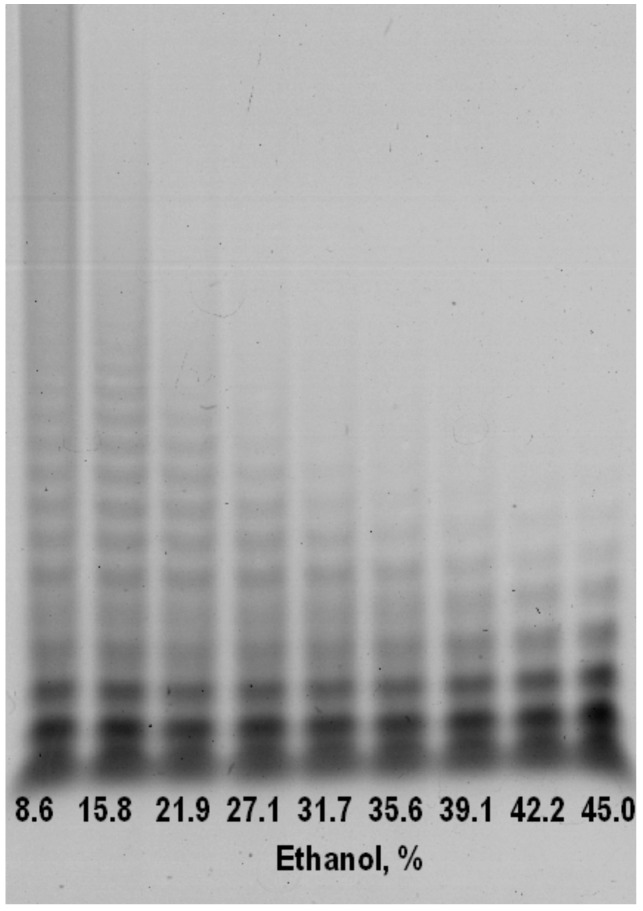
Products of action ALFA3 on mannuronate-enriched substrate precipitated by ethanol in ratios from 8.6 to 45.0% (low-molecular fraction). Numbers correspond to ethanol percentage in precipitation mixtures. Precipitation was run during 18 h at 4 °C.

Precipitation of alginate poly- and oligosaccharides with ethanol provides from incubation mixture is a controllable and simple method for isolating low-molecular fraction with a defined range of alginate oligosaccharides (AOS) (Figure 3). In comparison to anion-exchange chromatography this method has no need additional steps of desalting [11]. This diagram shows how to find necessary concentrations to obtain the fraction of purpose: first discard heavy fractions (39% of ethanol) in a residue and then precipitate the fraction of purpose with a higher concentration of ethanol (45% of ethanol). Isolating a low-molecular is an important step, because only AOS with low degree of polymerization can be potentially used as infusion drugs without a risk precipitation and thickening in blood. Furthermore, exactly AOS have shown the supreme therapeutical potential in compare with high-molecular fractions (alginate polysaccharides) [11].

**Figure 4:**
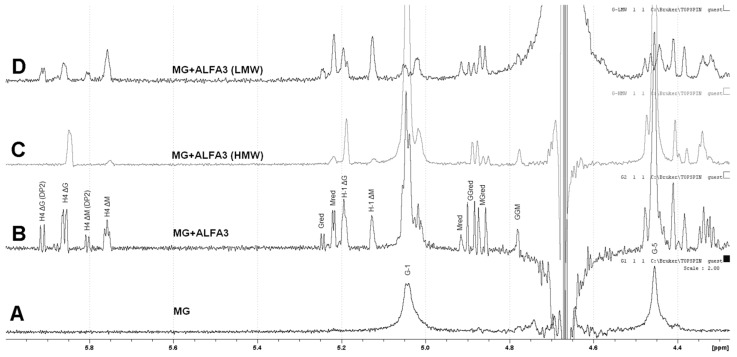
^1^H NMR analysis of products after action of ALFA3 on guluronate-enriched substrate. (**A**) mannuronate-enriched sodium alginate, (**B**) total products after action of ALFA3, (**C**) high-molecular weight fractions after action of ALFA3, and (**D**) low-molecular weight fractions after action of ALFA3.

^1^H NMR analysis of digestion products for guluronate-enriched substrate (M:G~1:2) showed that it was successfully hydrolyzed by ALFA3 (Figure 4A, B). The ^1^H spectrum of the hydrolyzed sample was compared to the previously reported data [20,21,22,23,24,25] to assign its NMR shifts. Since there were a visible accumulation of signals for residue ΔG’s H4 (5.87 and 5.95 ppm) and residue ΔM’s H4 (5.76 and 5.80 ppm), with the dominant signal from ΔG, the potential ALFA3-hydrolysed bonds were MM, MG and GM. The disproportionally low intensity of G red signal (5.25 ppm) relative to M red (5.22 ppm) allows concluding that MM, MG and GM but not GG bonds are cleaved. The differences in spectra of high-molecular-weight (HMW) (Figure 4C) and low-molecular-weight (LMW) (Figure 4D) fractions, separated by ethanol precipitation in ratio ethanol: reaction mixture 0.9:1, revealed that the HMW fraction contained mainly G-blocks with highly predominant M-links on reducing and ΔG links on non-reducing ends. In the LMW fraction, there were almost no G-blocks, with M- and G-links on reducing and ΔM and ΔG links on non-reducing ends. This effect was combined with a high percentage of oligosaccharides with DP 2 (Supplementary Figure S5).

**Figure 5:**
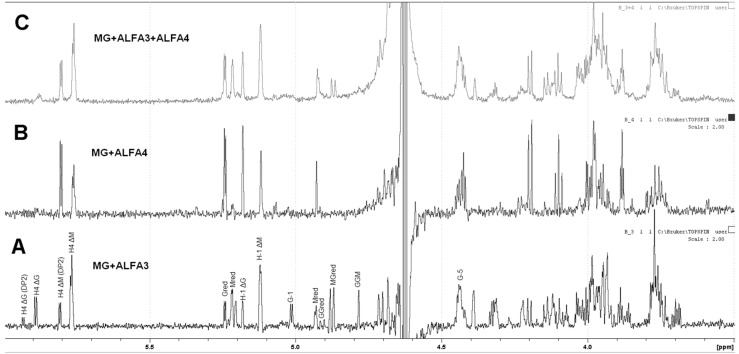
^1^H NMR analysis of action products of ALFA3 (**A**) and ALFA4 (**B**) on mannuronate-enriched substrate, as well as products of their joint action (**C**). M—sodium polymannuronate, MG—mannuronate-enriched sodium alginate, G—guluronate-enriched sodium alginate.

The products of ALFA3 and ALFA4 enzymes and the products of their combined action are quite different for the mannuronate-enriched substrate (M:G~3:1). For ALFA3 predominantly H4 ΔM signals (5.87 and 5.95 ppm) above H4 ΔG (5.76 and 5.80 ppm) are observed (Figure 5A), while ALFA4 produces only ΔM signals, predominantly dimers (Figure 5B). The combined action of ALFA3 and ALFA4 produces almost exclusively ΔM signals, but the share of dimers decreases (Figure 5C). This effect can be explained by ALFA4 producing fragments that are resistant to the ALFA3 attack.

Classification of alginate lyases into M-specific (EC 4.2.2.3), G-specific (EC 4.2.2.11), and alginate oligosaccharide lyases (4.2.2.26) prompted testing of the ALFA3 and ALFA4 substrate specificity on three different types of substrate (Table 1). Considering that polymannuronate- and mannuronate-enriched substrates were digested faster than the guluronate-enriched substrate, both enzymes were classified as M-specific (EC 4.2.2.3).

In the present study, the alginates and their products did not possess cytotoxic effect against melanoma SK-MEL-5, SK-MEL-28, and RPMI-7951 cell lines at concentrations up to 800 µg/mL.

**Figure 6:**
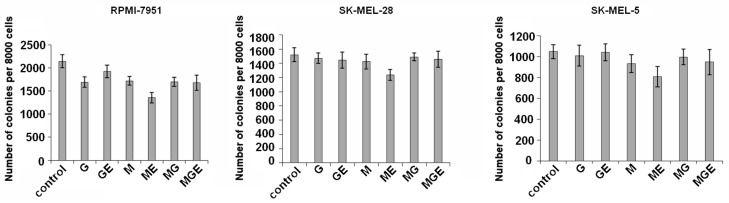
The effect of alginates and products of their digestion on colony formation of human cancer cells. All concentrations are 200 µg/mL.

Colony formation and growth of SK-MEL-5, SK-MEL-28, and RPMI-7951 cells was tested in the presence of non-toxic concentrations (200 µg/mL) of alginates and their derivatives using soft agar. The results showed that alginates and their products inhibited colony formation of these cells by less than 15%, although RPMI-7951 cells were more sensitive to the investigated compounds. Polymannuronate and products of its digestion by ALFA3 (i.e., mannuronate oligosaccharides) demonstrated higher activity and decreased the number of melanoma RPMI-7951 cells colonies by 20% and 37%, respectively (Figure 6).

Alginates and the products of polymannuronate, mannuronate-enriched and guluronate-enriched substrates digestion by ALFA3 (25–1000 µg/mL) did not affect the development rates and survivability of fertilized eggs of the sea urchin *Strongylocentrotus intermedius* (Figure S6). Both treated and control embryos reached pluteus stage in 36 h and stayed alive for up to 9–10 days. There was no significant difference in the morphology of treated and control embryos. 

## 3. Discussion

Two recombinant alginate lyases (ALFA3 and ALFA4) were cloned from the genome of marine bacterium *F. algae* KMM 3553^T^. Our results with different digestible substrates determined that ALFA3 is a bifunctional endolytic enzyme, while ALFA4 is an M-specific endolytic enzyme. Slight differences in substrate specificity allowed us to classify ALFA3 and ALFA4 as EC 4.2.2.3 (mannuronate-specific alginate lyase). These bifunctional alginate lyases form a sizable group in the pool of alginolytic enzymes. While there is no specific EC number assigned to this group, these enzymes can be classified as either mannuronate-specific (EC 4.2.2.3) or guluronate-specific (EC 4.2.2.11). Previously four alginate lyases from marine bacterium *Vibrios plendidus* 12B01 were isolated, and two of them could cleave the bond between M and G subunits, while the other two were guluronate-specific [20]. Recently, another bifunctional alginate lyase was isolated from marine bacterium *Flammeovirga* sp. strain MY04 [26] suggesting that the bifunctional mode of action in alginate-utilizing mechanism provided an obvious advantage for bacteria that could utilize a rich and reliable source of energy.

The relatively low molecular weight of these enzymes makes its recombinant production reliable and efficient due to fewer resources required for their synthesis. 

Alginic acids present up to 40% of brown algae dry weight with M/G ratio ranging from 0.21 in *Sargassum filipendula* to 2.28 in *Saccharina japonica* [27], so M-specific alginate lyase that can degrade even G-enriched substrate, provides its producer with a convenient tool for survival and adaptation.

Significantly, the optimum pH of ALFA3 was rather sharp for pure M- and G-enriched substrate, but it was much wider (6 to 9) for MG-substrate. This phenomenon can be explained by the differences in optimum conditions for digestion of MM and MG bonds, but this hypothesis requires further investigations.

Analyzing final digestion products of different substrates by ALFA4, we realized that the enzyme produced alginate oligosaccharides predominately from mannuronate-enriched and guluronate-enriched substrates but not from pure polymannuronate. Considering that ALFA4 has the highest activity with mannuronate-enriched substrate, a lower one with pure polymannuronate and relatively insignificant with guluronate-enriched one, it is possible to hypothesize that ALFA4 cleaves MM-bonds but needs some G-links for more effective substrate binding.

The initial substrates and products of their enzymatic digestion showed only moderate toxicity on human melanoma cells and almost no toxicity on fertilized eggs of sea urchin.

There was no difference if the substances were applied on fresh-fertilized eggs or blastulas (12 h after fertilization). In some cases, the cells or blastulas survived for 6–7 days longer than controls after treatment with products of alginate enzymatic digestion (both HMW and LMW in concentrations 0.5-1.0 mg/mL); this effect may be explained by the use of the alginates as a source of energy. If this is confirmed, the products of enzymatic digestion may be characterized as potentially non-toxic for healthy cells, opening the possibility for further long-term bioassays with healthy human cells.

In *F. algae* genome, all alginate lyases are in different gene clusters. This distribution suggests that algal alginic acids play a facultative role in the nutrition of this bacterium. 

In conclusion, in all cases of alginate substrate digestion with recombinant alginate lyases ALFA3 and ALFA4 of marine bacterium *F. algae*, there is a wide range of alginate oligosaccharides with DP from 1 to 20. This observation can be used for screening of products’ biological activity.

## 4. Materials and Methods

### 4.1. Genomic DNA Isolation and Construction of Expression Vector

*F. algae* KMM3553^T^ strain was grown as described by Ivanova et al. [16]. Its genome sequence (GenBank number PRJNA299442) was analyzed for the presence of alginate lyase genes using the RAST server [28]. The strain was obtained from Collection of Marine Microorganisms (KMM) in the G. B. Elyakov Pacific Institute of Bioorganic Chemistry (PIBOC), Vladivostok, Russia. Genomic DNA was isolated with AxyPrep™ Multisource Genomic DNA Purification Miniprep Kit (Axygen, New York, NY, USA) according to the manufacturer’s recommendations. The primers were designed using restriction-free cloning service [29] based on pColdII plasmid (Takara Bio, Kusatsu, Japan). The primers were designed:

ALFA3_dir 5’-CCGAGAACCTTTACTTCCAGGGGGAAGTTACAGATGATAATGCCGAC-3’

ALFA3_rev 5’-TCTTAGATTCTGTGCTTTTAAGCAGAGATTACCTATTATAAACTATGTTCGGTTTTTACCTTAT-3’

ALFA4_dir 5’- CCGAGAACCTTTACTTCCAGGGGGAAGATCCAGACGACATAGAAGAC-3’

ALFA4_rev 5’-TCTTAGATTCTGTGCTTTTAAGCAGAGATTACCTATTACTTTAAGTCTGCCGCCAA-3’

The PCR was done using Phusion HF polymerase (New England Biolabs, Ipswich, MA, USA). Thirty-five cycles of amplification were done to obtain the necessary amount of insert and vector. PCR reaction was controlled by electrophoresis in agarose gel. Sequence analysis was carried out using the NCBI BLAST [30]. Domain architectures were identified by InterProScan (http://www.ebi.ac.uk/interpro/) [31]. Multiple sequence alignment was done with the Clustal Omega server [32]. Competent cells were prepared using Mix & Go *E. coli* Transformation Kit & Buffer Set (Zymo Research, Irvine, CA, USA). Plasmids were cloned in *E. coli* XL10 Gold cells (Stratagene, San Diego, CA, USA) and extracted with QuantumPrep® Plasmid Miniprep Kit (Bio-Rad, Hercules, CA, USA). 

### 4.2. Expression and Purification of Recombinant ALFA3 and ALFA4

*E. coli* Arctic Express (DE3) (Agilent, Santa Clara, CA, USA) cells with plasmids pColdII-ALFA3 and pColdII-ALFA4 were grown overnight at 37 °C on LB plates containing 100 µg/mL ampicillin. A single colony was picked and grown at 200 rpm in LB with 100 µg/mL ampicillin at 37 °C for 12 h, then transferred to fresh LB with 100 µg/mL ampicillin. Cells were grown up to an optical density of approximately 0.6 OD600; expression was induced by IPTG (0.2 mM) and carried out for 16 h at 16 °C. Recombinant cells were collected by centrifugation at 6000 relative centrifugal forces (rcf) for 20 min at 4 °C, resuspended in 20 mM phosphate buffer (pH 7.4), disrupted by sonication, and centrifuged at 6000 rcf during 20 min at 4 °C. The resulting supernatant was applied to a 2 mL column of Ni-NTA agarose (General Electric, Chicago, IL, USA) pre-equilibrated with 10 mM sodium phosphate buffer (pH 7.4) with 0.5 M NaCl and 20 mM imidazole. Chromatography was performed with a linear gradient of 20–500 mM imidazole (total volume 40 mL), the flow rate of 0.5 mL/min. His-tag was removed by TEV-protease (Sigma-Aldrich, St. Louis, MO, USA). The enzyme was re-equilibrated in succinate buffer pH 6.0. This method produced pure alginate lyases ALFA3 and ALFA4 (single band by SDS-PAGE, see Figure S7).

### 4.3. Analytical Methods

Protein concentration was measured by the Bradford procedure with bovine serum albumin (BSA) as a standard [33]. The homogeneity and molecular weights of the enzymes were determined by SDS PAGE using the method of Laemmli [34] and 12% acrylamide gels. The Precision Plus Protein™ Standards (Bio-Rad, Hercules, CA, USA) was used as standard.

### 4.4. Electrophoresis of Carbohydrates (C-PAGE)

The substrates and products of their hydrolysis (15 μL, 4 mg/mL) were mixed with 5 μL of loading buffer containing a 20% solution of glycerol in water and 0.02% of phenol red. The samples (10 μL) were separated by electrophoresis through a 5% (w/v) stacking gel with 50 mM Tris–HCl buffer pH 6.8 and 27% (w/v) resolving polyacrylamide gel with 150 mM Tris–HCl buffer pH 8.8. The gel was 1 mm thick. The gel was stained with 0.02% O-toluidine blue (Sigma-Aldrich, St. Louis, MO, USA) and 0.3% alcian blue in EtOH, AcOH, and H_2_O with a volume ratio of 2:1:1. The gel images were visualized using a calibrated densitometer DS-800 (Bio-Rad).

### 4.5. Substrates and Enzyme Assay

Sodium polymannuronate was isolated from brown alga *Saccharina cichorioides* in the Laboratory of Enzyme Chemistry, G.B. Elyakov Pacific Institute of Bioorganic Chemistry, Russia PIBOC FEB RAS. Guluronate-enriched sodium alginate (BDH, UK, M:G~1:2), and mannuronate-enriched sodium alginate (Sigma-Aldrich, St. Louis, MO, USA, M:G~3:1) were also used.

The activity of alginate lyase was deduced from the increase in reducing sugars [35]. The incubated mixture contained 100 µL of the enzyme, 200 µL of sodium alginate solution (4 mg/mL in 25 mM succinic buffer, рН 6.0). The incubation was for 1 h at 37 °C to cleave approximately 10% of the substrate. Unit of activity is determined as the amount of the enzyme that catalyzed the formation of 1 µmole of monomer per min. Enzyme and substrate controls were used to exclude false positives. All experiments were done at least three times.

### 4.6. Enzyme Characterization

The optimum pH values for ALFA3 and ALFA4 were determined by the standard activity assay for 1 h at 37 °C using 0.2 M citrate-phosphate buffer in the range of pH 2.0–10.0. The reaction mixture contained 100 µL of the enzyme solution, 200 µL of the substrate solution (4 mg/mL), and 300 µL of a buffer with various pH.

The optimum temperature was determined by the standard activity assay for 1 h at temperatures between 25 and 55 °C with 5 °C intervals and the concentration of reducing sugars was measured.

Thermal stability was estimated by measuring the residual activity by standard assay after pre-incubation of the enzyme at temperatures between 10 and 70 °C for 1 h. 

Km was calculated from the Lineweaver-Burk plot using data collected by measuring the rate of mannuronate-enriched sodium alginate hydrolysis under the standard assay conditions for 1 h at 37 °C from 0.025 to 2.0 mg/mL.

### 4.7. Enzyme Classification

Classification of the enzymes by amino acid sequences was done using InterPro [36]. Classification of the enzymes by the type of activity was done according to the enzyme activity on different substrates: polymannuronate, mannuronate-enriched alginate, and guluronate-enriched alginate (see the Section 4.5).

### 4.8. Obtaining the Products of Exhaustive Hydrolysis by ALFA3 and ALFA4

For exhaustive hydrolysis, 100 µL of enzyme solution (1 mg/mL) was added to a solution of different sodium alginate substrates (4 mg/mL, 25 mL), and the mixture was incubated at 37 °C. Aliquots (50 μL) were taken at 0, 0.5, 1.5, 3.5, 6, 24, 48, 72, and 96 h, and the reaction was terminated by heating in a solid-state thermostat (DNA Technology, Russia) for 1 min at 99 °C. Products were analyzed by C-PAGE. After 96 h, the reaction mixture was concentrated by rotary evaporator (IKA, Staufen, Germany) at 50 °C and lyophilized.

Fractionation of alginate oligosaccharides was performed with ethanol precipitation. After the end of reaction, the enzyme was inactivated by heating the reaction mixture at 90 °C during 1 h and then cooling it to the room temperature. Ninety-five percent ethanol was added to aliquots of inactivated reaction mixture in ration (ethanol:mixture) from 0.1:1 to 0.9:1, so the final concentrations of ethanol were from 8.6 to 45%. The aliquots were stirred well and then incubated at 4 °C for 18 h. After incubation, the aliquots were centrifuged at 9000 rcf during 30 min, and the supernatants were collected, concentrated by rotary evaporator (IKA, Germany) at 50 °C, lyophilized, resuspended in water (up to 4 mg/mL), and analyzed by C-PAGE.

^1^H NMR spectra were recorded using a Bruker DRX 500 spectrometer 500 MHz (Bruker, Germany). There were taken the spectra of the native substrates and the reaction mixtures (96h of reaction) (all samples by 10 mg). The reactions of hydrolysis (4 mg/mL of substrate, 0.1 U/mL of enzyme) were run in 25 mM succinic buffer pH 6.0 at 37 °C, so after resuspension, pH was close to 6.0. The products of the exhaustive hydrolysis were freeze-dried, resuspended in D_2_O, and the spectra were taken at 35 °C. The volume of the sample was 0.5 mL.

### 4.9. Bioassays with Human Melanoma Cells

#### 4.9.1. Chemicals and Cell Lines

Dulbecco’s Modified Eagle Medium (DMEM), phosphate-buffered saline (PBS), l-glutamine, penicillin-streptomycin solution, trypsin, fetal bovine serum (FBS), sodium hydrocarbonate (NaHCO_3_), and agar were purchased from Biolot (Moscow, Russia).

Human melanoma SK-MEL-5 (ATCC® no. HTB-70™), SK-MEL-28 (ATCC® no.HTB-72™), RPMI-7951 (ATCC® no. HTB-66™) cell lines were purchased from the American Type Culture Collection (ATCC, Manassas, VA, USA).

#### 4.9.2. Cells Cultivation

Human melanoma SK-MEL-5, SK-MEL-28, and RPMI-7951 cell lines were cultured in DMEM medium, supplemented with 10% fetal bovine serum (FBS), 200 mM l-glutamine, and penicillin-streptomycin solution. The cell cultures were maintained at 37 °C in a humidified atmosphere containing 5% CO_2_.

#### 4.9.3. Cytotoxicity Assay

3-(4,5-dimethylthiazol-2-yl)-5-(3-carboxymethoxyphenyl)-2-(4-sulfophenyl)-2H-tetrazolium (MTS) assay was used as the indicator of cell viability determined by mitochondrial-dependent reduction of formazan. In brief, cells were seeded at a density of 1.0 × 10^4^ cells per well in 200 µL of the complete medium in 96-well plates. After 24 h incubation, the attached cells were treated with various concentrations of alginates and its products (100, 200, 400, 800 µg/mL), while the control was mock-treated with the DMEM medium only. Cells were cultured for an additional 24 h at 37 °C in 5% CO_2_ incubator. After incubation MTS reagent (20 µL) was added to each well, and cells were incubated for 3 h at 37 °C in 5% CO_2_. Absorbance was measured at 490/630 nm by a microplate reader Power Wave XS (Bio-Tek, Winooski, VT, USA). All tests were carried out in triplicate.

#### 4.9.4. Anchorage-Independent Colony Formation Assay

SK-MEL-5 (A), SK-MEL-28 (B), and RPMI-7951 cells (2.4 × 10^4^/mL) were seeded in six-well plate with or without alginates and its products (200 µg/mL) in 1 mL of 0.3% Basal Medium Eagle (BME) agar containing 10% FBS, 2 mM l-glutamine, and 25 µg/mL gentamicin. The cultures were maintained at 37 °C in a 5% CO_2_ incubator for 14 days, and the cell’s colonies were scored using a microscope Motic AE 20 (Motic, Xiamen, China) and the Motic Image Plus computer program.

### 4.10. Bioassays with Sea Urchin Embryos

Sea urchins *Strongylocentrotus intermedius* were collected in Troitsa Bay, Sea of Japan, Russia, in September 2019 as described [37]. In brief, the eggs and developing embryos of the sea urchin were used at a concentration of 2500 cells per mL of seawater. The concentrations of substances were between 25 and 1000 µg/mL. The substances were applied to fertilized eggs at the zygote stage and actively mobile embryos at the blastula stage. The development was observed with Motic AE21 microscope and Levenhuk C-series camera (Levenhuk, Tampa, FL, USA).

### 4.11. Statistical Assay

All assays were performed at least in triplicate. The results are expressed as the mean ± standard deviation (SD). A Student’s t-test was used to evaluate the data with the following significance levels: * p < 0.05, ** p < 0.01, *** p < 0.001.

## Figures and Tables

**Table 1:** Properties of recombinant alginate lyases of *Formosa algae.*

Enzyme	Molecular Weight, kDa	Specific Activity, U/mg			EC Number	Structure Family
		PolyM	G-enriched	M-enriched		
ALFA3	33.8	21.3	18.7	21.5	4.2.2.3	PL7
ALFA4	89.8	1.3	0.2	2.2	4.2.2.3	PL6

## Supplementary Materials

The following are available online at https://www.mdpi.com/1660-3397/18/2/130/s1.
