# Supplementary material for: Two New Alginate Lyases of PL7 and PL6 Families from Polysaccharide-Degrading Bacterium Formosa algae KMM 3553T: Structure, Properties, and Products Analysis
Source: Mar Drugs. 2020 Feb 24;18(2):130. doi: 10.3390/md18020130 (PMC7074159; doi:10.3390/md18020130)
Supplement: Supplementary file 1 [file marinedrugs-18-00130-s001.pdf]

marine drugs

MDPI

Supplementary materials

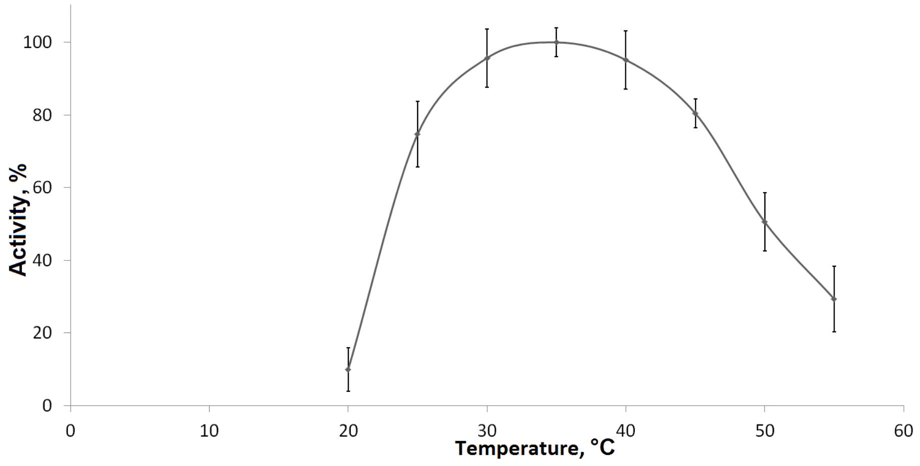

Figure S1. Optimal temperature evaluation for ALFA3. Standard deviations are given.

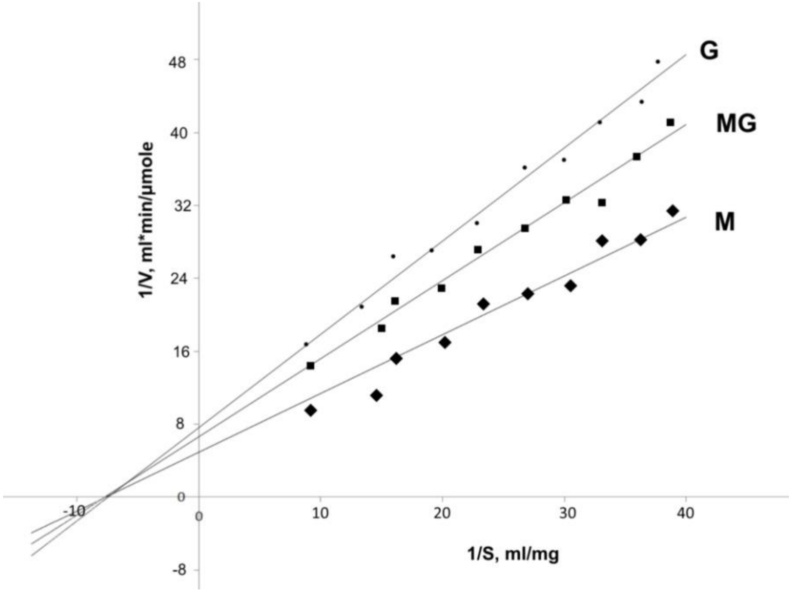

Figure S2. Lineweaver-Burk plot, visualizing Km and Vmax values for ALFA3 on three different substrates, used in the experiment.

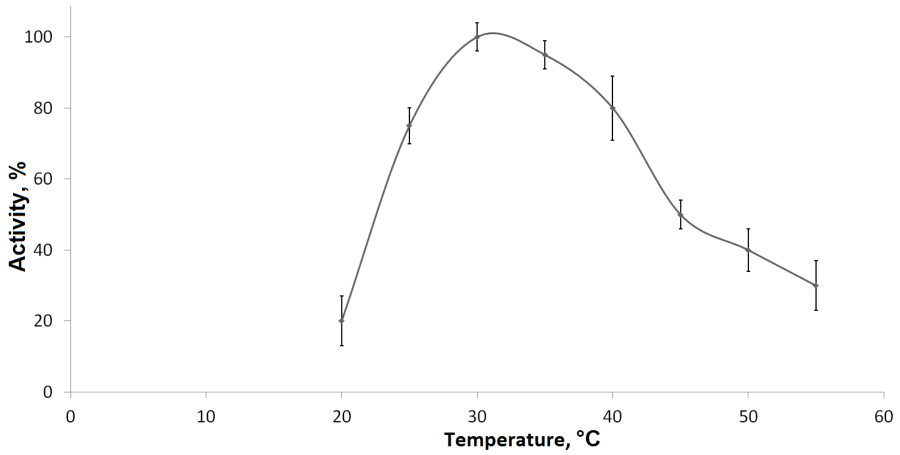

Figure S3. Optimal temperature evaluation for ALFA4. Standard deviations are given.

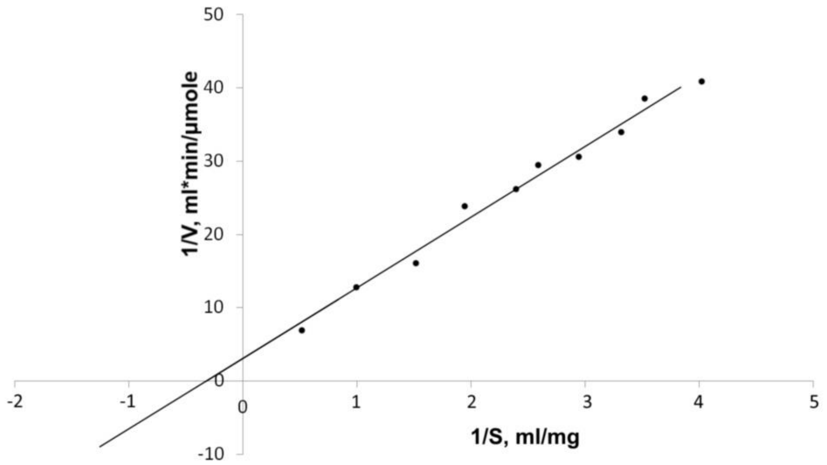

Figure S4. Lineweaver-Burk plot, visualizing Km and Vmax values for ALFA4 on mannuronare-enriched substrate.

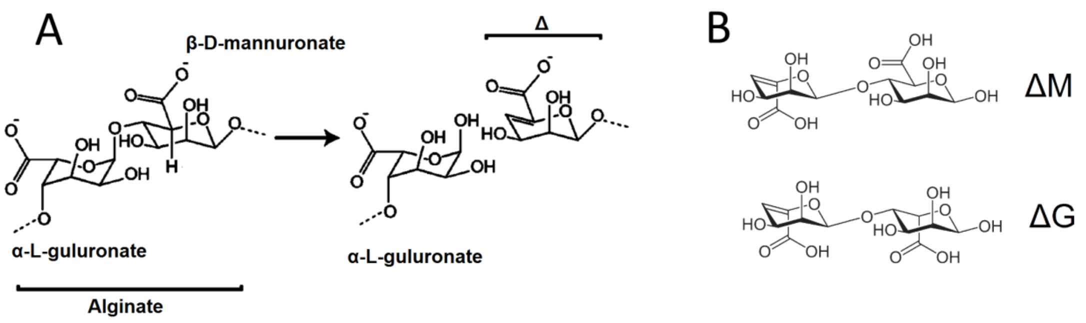

Figure S5. Typical scheme of alginate hydrolysis by alginate lyase (A), formulas of alginate disaccharides ΔM and ΔG – the patters of the variety of enzymatically produced alginate oligosaccharides (B).

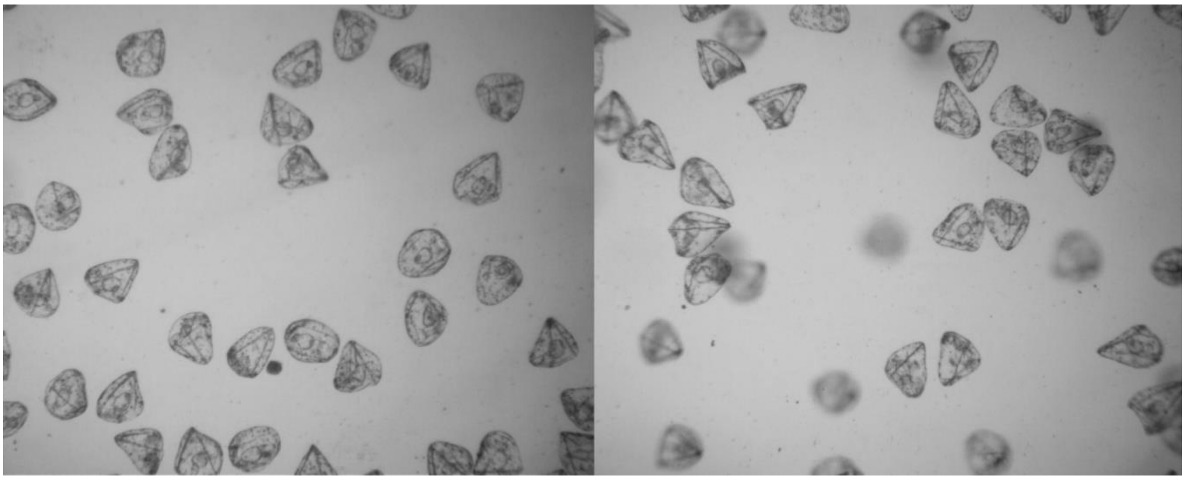

Figure S6. Sea urchin *Strongylocentrotus intermedius* embryos 36h after fertilisation. Left – typical picture of plutei after treatment, right – control. There is no significant difference between treated and control organisms that can be assumed as evidence of biological safety of alginates and alginate oligosaccharides.

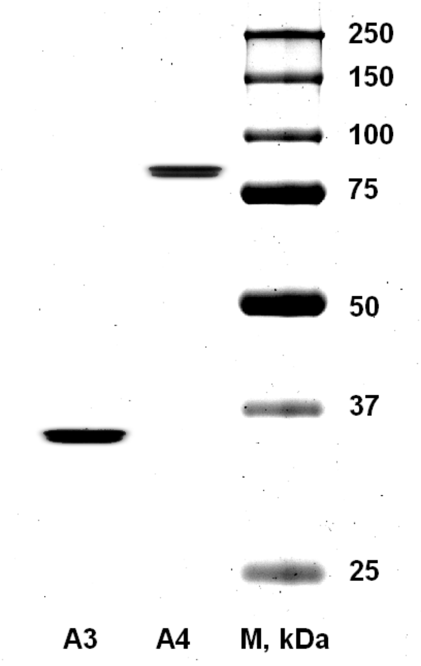

Figure S7. Electrophoresis gel photograph showing purity and molecular masses of ALFA3 (mentioned as A3) and ALFA4 (mentioned as A4) alginate lyases.

© 2020 by the authors. Submitted for possible open access publication under the terms and conditions of the Creative Commons Attribution (CC BY) license (http://creativecommons.org/licenses/by/4.0/).
